# LCAT: an isoform-sensitive error correction for transcriptome sequencing long reads

**DOI:** 10.3389/fgene.2023.1166975

**Published:** 2023-05-24

**Authors:** Wufei Zhu, Xingyu Liao

**Affiliations:** ^1^ Department of Endocrinology, Yichang Central People’s Hospital, The First College of Clinical Medical Science, China Three Gorges University, Yichang, China; ^2^ Computer, Electrical and Mathematical Sciences, and Engineering Division, King Abdullah University of Science and Technology (KAUST), Thuwal, Saudi Arabia

**Keywords:** RNA, full-length transcriptome, third-generation sequencing, error correction, isoform diversity keeping

## Abstract

As the carrier of genetic information, RNA carries the information from genes to proteins. Transcriptome sequencing technology is an important way to obtain transcriptome sequences, and it is also the basis for transcriptome research. With the development of third-generation sequencing, long reads can cover full-length transcripts and reflect the composition of different isoforms. However, the high error rate of third-generation sequencing affects the accuracy of long reads and downstream analysis. The current error correction methods seldom consider the existence of different isoforms in RNA, which makes the diversity of isoforms a serious loss. Here, we introduce LCAT (long-read error correction algorithm for transcriptome sequencing data), a wrapper algorithm of MECAT, to reduce the loss of isoform diversity while keeping MECAT’s error correction performance. The experimental results show that LCAT can not only improve the quality of transcriptome sequencing long reads but also retain the diversity of isoforms.

## 1 Introduction

Ribonucleic acid (RNA) is responsible for transferring genetic information from DNA to proteins. The genetic information contained in RNA plays an important role in the encoding, decoding, expression, and regulation of many biological functions. Unlike the double-stranded form of DNA, RNA is usually present in cells and some viruses in a single-stranded form as a carrier of genetic information. RNA splicing occurs during the conversion of pre-RNA to mRNA. RNA can combine exons in various ways through alternative splicing events at different developmental stages or in different tissues of the same organism. Transcripts formed by alternative splicing are called isoforms. Alternative splicing widely exists in non-prokaryotes. It has been shown that alternative splicing is present in more than 95% of multiple exon genes in the human genome (Wang et al., 2008). Alternative splicing makes transcripts and proteins more complex and variable in function and structure, which is an important regulatory mechanism for organisms. In addition, since various isoforms are produced under specific conditions and tissues, it is possible to associate with the corresponding tissues, time, or specific environment. Studies have shown that specific alternative splicing is correlated with various diseases (Deonovic et al., 2017); thus, the research on alternative splicing is greatly meaningful. Transcriptome sequencing is an important technique for obtaining complete RNA sequences and is also the basis of many transcriptome studies. Using the reads obtained by sequencing technology, many transcriptome studies can be performed, such as quantifying gene expression levels, identifying alternative splicing sites, identifying new transcripts, and quantifying isoform expression levels. Therefore, obtaining high-quality complete transcripts has become the basis of research in the transcriptome.

In recent years, third-generation sequencing technologies represented by PacBio (Rhoads and Au, 2015; Gochez et al., 2018; Kim et al., 2018) and Nanopore (Senol Cali et al., 2019) have developed rapidly. Third-generation sequencing reads have the characteristics of long fragment lengths and a high error rate. For example, the initial average error rate and the fragment length of PacBio long reads are approximately 15% and 1.5 kb (Quail et al., 2012), respectively. With the continuous development of Nanopore sequencing technology, the error rate and fragment length are also constantly changing. For instance, the average fragment length of Nanopore long reads can reach hundreds of kb, and the average error rate has also been reduced from 15% of 1D to 13% of 2D and 5% of 1D2 technologies (Wang et al., 2021; Jain et al., 2022; Svrzikapa and Boyanapalli, 2022; Gao et al., 2023; Kovaka et al., 2023). The average length of full-length transcripts is approximately 1.5 kb. Therefore, some of the long reads can cover the full-length transcripts without assembly, which reduces the problems and challenges introduced by transcriptome assembly (Yuwen et al., 2020). In addition, third-generation sequencing long reads can better describe the different combinations of exons and introns to achieve the purpose of identifying isoforms. Long reads have been increasingly used in transcriptome research due to the development of third-generation sequencing technologies and their significant advantages.

At present, there have been many studies that perform isoform annotation based on full-length transcripts obtained by third-generation sequencing. For example, Thomas et al. (2014) collected and purified RNA from chicken hearts and sequenced the cDNA library using third-generation sequencing technology. Afterward, Aken et al. (2016) mapped the sequencing reads to the Ensembl annotation library and found thousands of transcript isoforms. In this study, hundreds of transcripts have been identified, which improved the quality of the biological annotation library. However, it also reflects the limitations of long reads, with over 90% of reads covering only 42% of the annotation set. The high error rate of the third-generation sequencing technique limits the accuracy of long reads in transcriptome studies (Weirather et al., 2017), especially for the accurate detection of exon boundaries and the identification of isoforms with high similarity. Unlike second-generation reads, where the error rate is within 1% and the majority of errors are dominated by mismatches (Xingyu et al., 2019), the randomness of sequencing errors in traditional PacBio and Nanopore long reads (excluding HiFi and CCS reads) consists of more indels than mismatches (Ye et al., 2016), and their error rate is much higher than that of the former techniques. Third-generation sequencing, such as traditional PacBio and Nanopore, has not only brought unprecedented opportunities for the acquisition of full-length transcripts but also brought great challenges to downstream analysis, such as sequence alignment, isoform detection, and intron–exon boundary identification, increased the complexity of biological computing, and affected the accuracy of analysis results.

Error correction of third-generation sequencing reads is fundamental for improving the quality of transcriptome. At present, there are three categories of methods for long-read error correction, namely, biological error correction, hybrid error correction, and self-error correction. Circular consensus sequencing (CCS) is a biological error correction technology that can reduce the error rate of PacBio reads (Travers et al., 2010). In 2019, the accuracy of CCS reads exceeded 99% (Wenger et al., 2019). The hybrid error correction method utilizes short reads with low production cost and high throughput to correct and compensate for the third-generation sequencing long reads with low coverage and high error rate. In recent years, several hybrid error correction tools have been developed for third-generation sequencing reads, such as LSCplus (Hu et al., 2016), proovread (Hackl et al., 2014), and LoRDEC (Salmela and Rivals, 2014). The self-error correction method is the most potential of the current three kinds of methods that corrects long reads by finding the overlapping relationship between them without any other additional data.

Although the existing self-error correction methods are all designed for DNA sequencing data, these tools can also achieve error correction effects on RNA sequencing data. For example, FALCON (Chin et al., 2016), Canu (Koren et al., 2017), and MECAT (Xiao et al., 2017) are three famous algorithms for self-error correction and genome assembly with third-generation sequencing reads. Among them, FALCON uses DALIGNER (Myers, 2014) to align all the long reads with each other, removes high-frequency *k-mers* during the alignment to reduce the effect of repeating regions, and uses FALCON-sense to find consensus sequences. This method simply neglects the high repetition of the *k-mers*, which may lead to the loss of correct overlapping information and reduce the accuracy of alignment. Due to the repetitive feature of the gene, the number of matched *k-mers* does not correspond to the length of the overlap and thus cannot be used directly as selection criteria for higher quality, more reliable matches. Local alignment is still required to screen many candidate matches, which also significantly increases the computational cost of error correction for the third generation of reads.

Canu constructs a similar read hash table at the alignment stage and obtains the overlapping relationship of reads by computing the shared *k-mer*, which employs a term frequency–inverse document frequency (tf–idf) (Kim and Gil, 2019) algorithm to weight the *k-mers* to reduce the impact of repeated *k-mer* matches. The FALCON-sense approach is also used by Canu in the consensus-finding step. However, Canu performs *k-mer* matching without considering the order of *k-mer* alignment and relative position relationships, so there are still many overmatches.

MECAT finds well-matched reads and best-matched base positions through local alignment and uses a pseudo-linear alignment scoring algorithm to filter out excessive alignment sequences, which uses a distance difference factor (DDF) to score matching *k-mers* in two steps. The score of the matched *k-mer* is determined by DDF, which can represent the matching and distance relationship between *k-mers*, thereby determining the alignment score between reads. After filtering through the DDF score, the candidate reads are reduced by 50%–70%, of which the quality is high. MECAT combines FALCON-sense and DAGCon in the consensus phase. For simple areas, MECAT uses a list voting method to find consensus while using a construct graph method to find paths with the largest weight as the consensus sequence for complex areas. Compared with other error correction methods, DDF scores filtering, and alignment is 2–3 times faster. Therefore, MECAT is also the fastest error correction tool available.

FLAS (Bao et al., 2019) is a self-error correction method developed based on MECAT. Compared with MECAT, FLAS mainly made two improvements. First, FLAS finds additional matches based on MECAT alignment and removes false match reads, which constructs a string graph of the MECAT match result and uses the Bron–Kerbosch (David et al., 2010) algorithm to find the largest clique in the graph. Second, FLAS uses the long reads that have been modified to perform a second error correction on the unmodified reads, thereby further improving the throughput of the results. LoRMA (Salmela et al., 2017) constructs de Bruijn graphs dynamically during the error correction process. Self-correction represents the future development direction of third-generation sequencing long read error correction. However, they are all designed for DNA third-generation sequencing reads. Although these methods can also be used to correct RNA long reads, there are still some limitations.

Some studies have applied DNA self-correction methods to RNA sequencing reads, and the following conclusions were obtained (Lima et al., 2020): first, the DNA self-correction tool can be used for RNA error correction, which can basically complete the improvement in the base error correction metrics, maintain a certain throughput, and improve the mapping rate of the corrected reads. Second, DNA self-correction tools for correcting RNA readings may lose isomer diversity and tend to bias the major isomers in the correction process. Based on the limitations analysis of the aforementioned DNA self-error correction methods, we proposed a new self-error correction algorithm based on MECAT, called LCAT, for the error correction of transcripts obtained through third-generation sequencing. The proposed method can effectively solve the problem of reducing the diversity of isoforms in the process of error correction and is more suitable for transcriptome data than other existing self-correction tools.

## 2 Materials and methods

**FIGURE 1:**
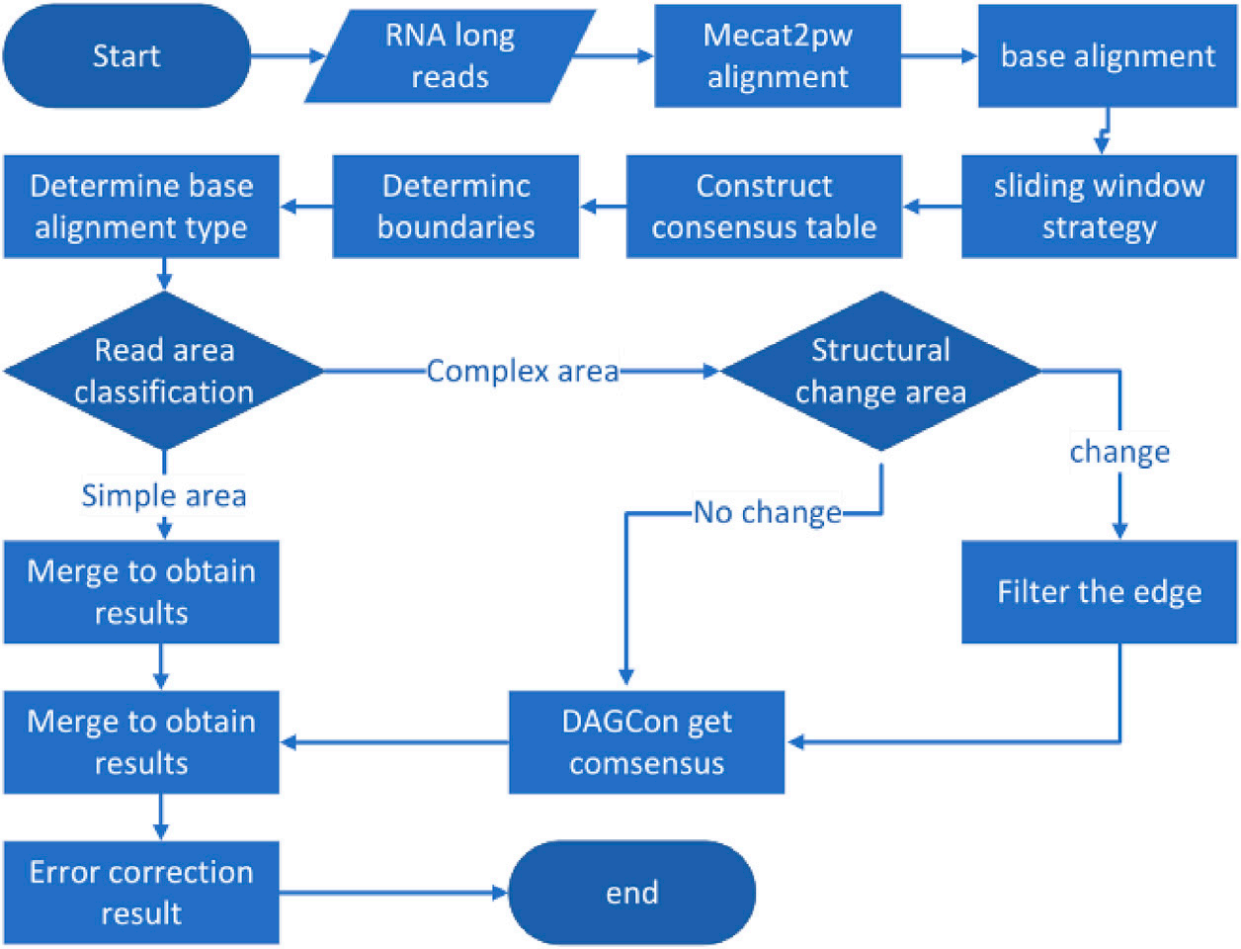
Pipeline of LCAT. Parallelogram represents the input data. Rectangles represent data processing steps. Diamonds represent judgments in the direction of processing execution.

LCAT is designed to preserve the structural characteristics of transcriptome isoforms based on MECAT. Reads aligned using MECAT assumes the same form of composition, but transcripts under the same gene can be composed in multiple ways. LCAT adopts the sliding window strategy to filter the read areas with low similarity after the alignment step to ensure that the areas aligned in the consensus stage come from the same exons. LCAT consists of the following steps: read alignment, base alignment, sliding window strategy, determining base alignment type, read partitioning, and consensus. The flow chart of LCAT is shown in Figure 1. The detailed principle of LCAT is described in the following sections.

### 2.1 Alignment and sliding window-based strategies

#### 2.1.1 Base alignment

**FIGURE 2:**
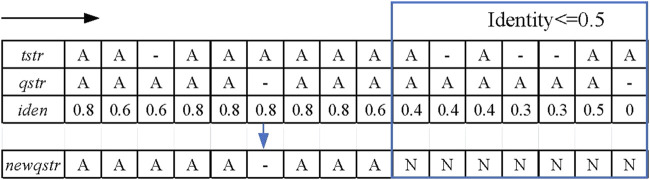
Schematic diagram of the sliding window strategy. The black arrow indicates the moving direction of the sliding window, and the framed part is the alignment area whose identity is not greater than 0.5.

After the template read is obtained and the candidate reads have been mapped to the template read, LCAT will traverse template read and candidate reads from the starting base. If the current traversed bases are the same, the two bases at this point are aligned. If the current position of the candidate read is different from the template read and the sequence of the next position is the same, a mismatch error occurs at that base. Otherwise, LCAT uses *'-'* to cross the base, which is called the insertion or deletion error. Next, LCAT normalizes the results of the base alignment by splitting the mismatch errors into insertion errors and deletion errors. In this way, an accurate alignment result between the template read and each candidate read can be obtained. Figure 2 shows the results of the base alignment. *‘A'* stands for the base, and “*tstr*” and “*qstr*” stand for the results of the base alignment of the template and candidate reads, respectively.

#### 2.1.2 Sliding window strategy

In the sliding window strategy step, the alignment sequence “*qstr*” of the candidate reads is first copied to a new sequence “*newqstr*.” Each position of the template reads is traversed, as well as the candidate reads in the alignment result, and the identity of the current position is calculated. The formula for calculating the identity is shown in formula (1).

$$identify=\frac{match\_base\_num}{window\_length},$$ (1)

where “*match_base_num*” represents the number of matched bases in the sliding window and “*window*_*length*” indicates the length of the sliding window. When the identity is less than the identity threshold, this position in *‘newqstr’* is replaced with the character “N,” which is a potential anomaly. In the LCAT tool, the user can manually set the length of the sliding window and identity threshold. Figure 2 shows an example of the sliding window strategy for processing base alignment results. In this example, the identity threshold is set to 0.5, the sliding window length is set to 5, and the red rectangle is the area of potential anomalies.

### 2.2 Determining base alignment type

#### 2.2.1 Construction of the consensus table

**FIGURE 3:**
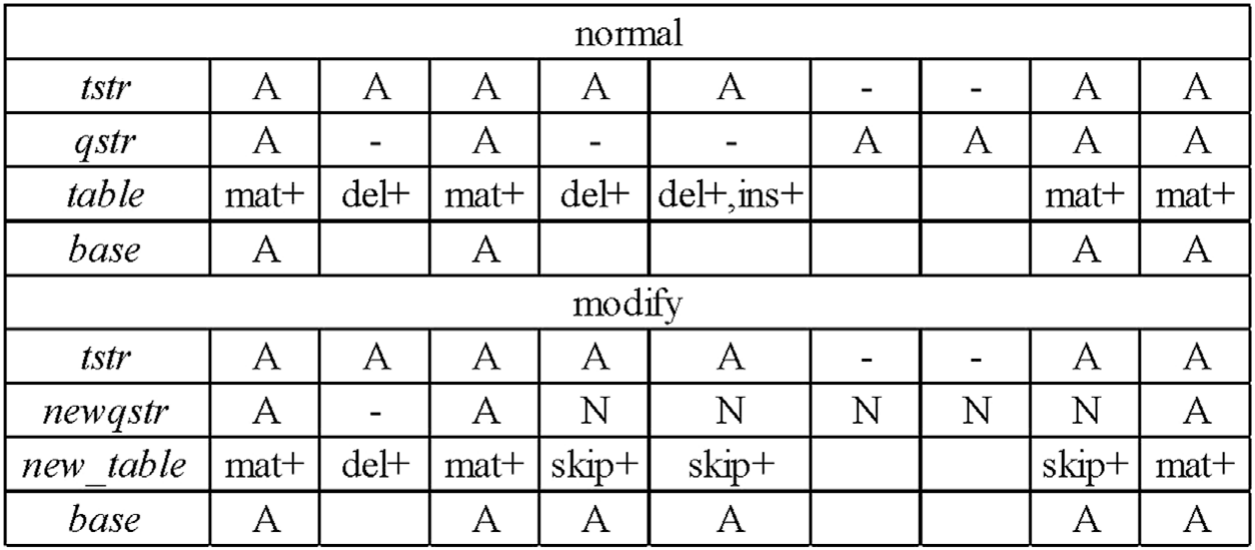
Construction of the consensus table.

During the process of constructing the consensus table, LCAT adds new statistics to the number of skips and improves the construction process of the consensus table. According to the base alignment result between each candidate read and the template read, a sequence consensus table of each template read is obtained. Figure 3 shows the consensus table construction process of the template read “*tstr*” and one of its candidate read “*qstr*.” According to the alignment, the current match type at each position, whether the four numbers (match, insert, delete, and skip) need to be increased, and the base of this position are determined.

Figure 3 shows that a “*normal*” consensus table construction process is performed based on the template read “*tstr*” and the candidate read “*qstr*” to obtain a consensus table. According to the template read “*tstr*” and the new candidate read “*newqstr*” processed by the sliding window strategy, the construction process of the “*modify*” consensus table is determined, and the consensus table “*new_table*” is obtained.

#### 2.2.2 Determination of the left and right boundaries

**FIGURE 4:**
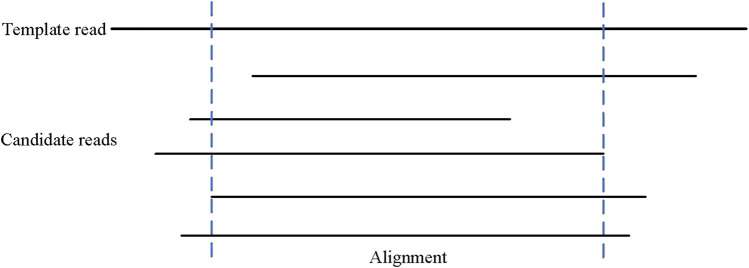
Determination of the left and right boundaries.

After traversing all candidate reads to obtain complete consensus tables of template reads, LCAT calculates the left and right boundaries of each template read. LCAT obtains the left and right boundaries by filtering the area where the coverage of the position is lower than the minimum coverage threshold. The filtering condition is shown in formula (2).

match+insert+skip≥min⁡_coverage, (2)

where “*match*” represents the number of matched bases, “*insert*” represents the number of inserted bases, “*skip*” indicates the number of skipped bases, and “*min_coverage*” indicates the minimum coverage. Unlike the boundary filtering condition of MECAT, LCAT increases the statistics of the skip number and regards the sum of insertion, match, and skip numbers at a position as the coverage number of that position. Only when the number of base coverage is not less than the minimum coverage, consensus error correction can be taken for this base. The results of determining the left and right boundaries are shown in Figure 4. In the figure, the minimum coverage is set to 4, and the areas within the dotted lines are the effective areas for final error correction.

#### 2.2.3 Determining base alignment type

**FIGURE 5:**
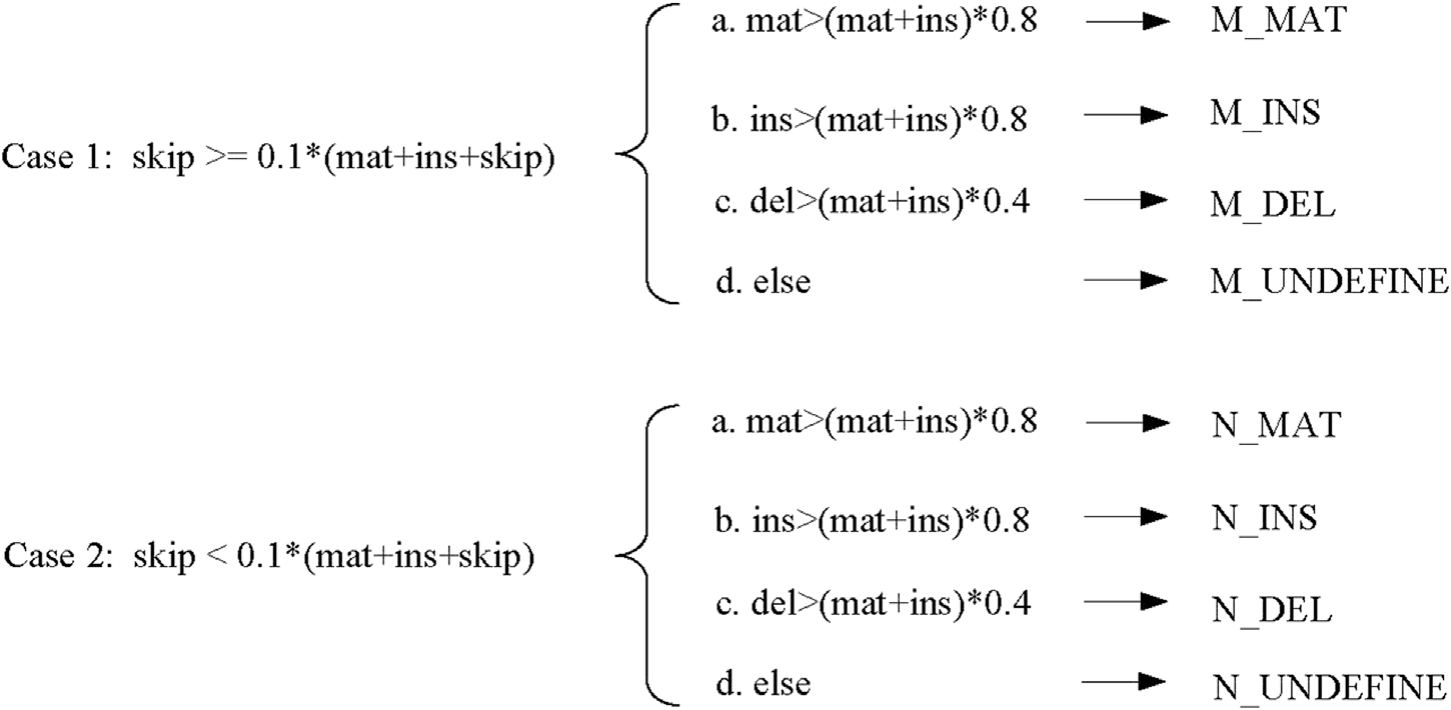
Base alignment type determination.

When determining the base alignment type at each position of the template read, LCAT first judges whether the character “*N*” to be replaced in the sliding window strategy should be retained. LCAT judges whether the regions with a non-zero amount of skip in the consensus table are regions with different structures. Here, LCAT uses the coverage to determine whether to retain “*N*.” If the number of skips at this position is greater than the total coverage * scov, then this position is a region where the structure appears different, i.e., the alignment regions are different exons. For such areas, LCAT uses “*new_table*” generated by “*newqstr*” in the step of constructing the consensus table to determine the base alignment type of this position, as shown in Case 1 in Figure 5. Otherwise, LCAT uses “*table*” generated by “*qstr*” in the step of constructing the consensus table to determine the base alignment type of this position, as shown in Case 2 in Figure 5; *scov* is a coefficient used to judge whether different structures appear in the area, and the user can set it in LCAT. Then, LCAT determines the base alignment type of each position.

There are four types of base alignment: match (MAT), insert (INS), delete (DEL), and undefined (UNDEFINE). In the match-type prefix, “*M*” represents that different regions of the structure appear, while “*N*” represents that no different regions of the structure appear. The specific judgment conditions are shown in Figure 5. In the figure, *scov* takes the default value of 0.1.

### 2.3 Read area classification and consensus

#### 2.3.1 Distinguishing the simple and complex areas

**FIGURE 6:**
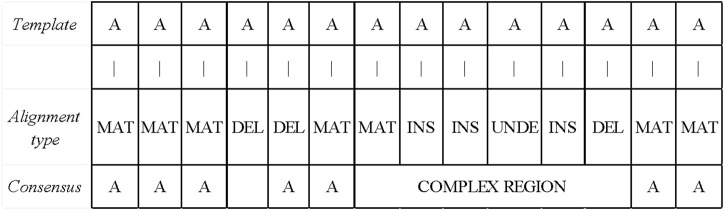
Read area division and simple area correction. If the alignment type is DEL between the two MAT types, this interval is also a simple area. If there is a non-MAT and non-DEL types between the two MAT types, the interval is a complex area.

After obtaining the base alignment type of the template read, LCAT divides the read into the simple and complex areas according to the distribution of the alignment type. The simple area is the area in which the alignment type is MAT. If the alignment type is DEL between the two MAT types, this interval is also a simple area. If there is a non-MAT and non-DEL types between the two MAT types, the interval is a complex area. The read area classification process is shown in Figure 6. Different consensus algorithms are used to correct errors according to different regions.

#### 2.3.2 Error correction for reads in the simple area

LCAT uses FALCON-sense algorithm to get consensus for simple area reads. The position of the alignment-type MAT is still the base of the position after error correction. If the position with the alignment-type DEL is in a non-complex area, LCAT will delete the base of this position, as shown in Figure 6. The FALCON-sense algorithm counts different types of bases. The speed of FALCON-sense is faster, while the accuracy is not high, which is suitable for simple area error correction.

#### 2.3.3 Error correction for reads in the complex area

When correcting errors in a complex area, LCAT first judges whether the area is a structural change area. The judgment is based on whether the character “*N*” appears in the base alignment sequence. If it appears, there are different exon structures in the region. LCAT deletes the edge containing “*N*” and calculates consensus based on the constructing graph. On the contrary, if the character “*N*” does not appear in the base alignment sequence, LCAT uses the graph method to get consensus directly.

**FIGURE 7:**
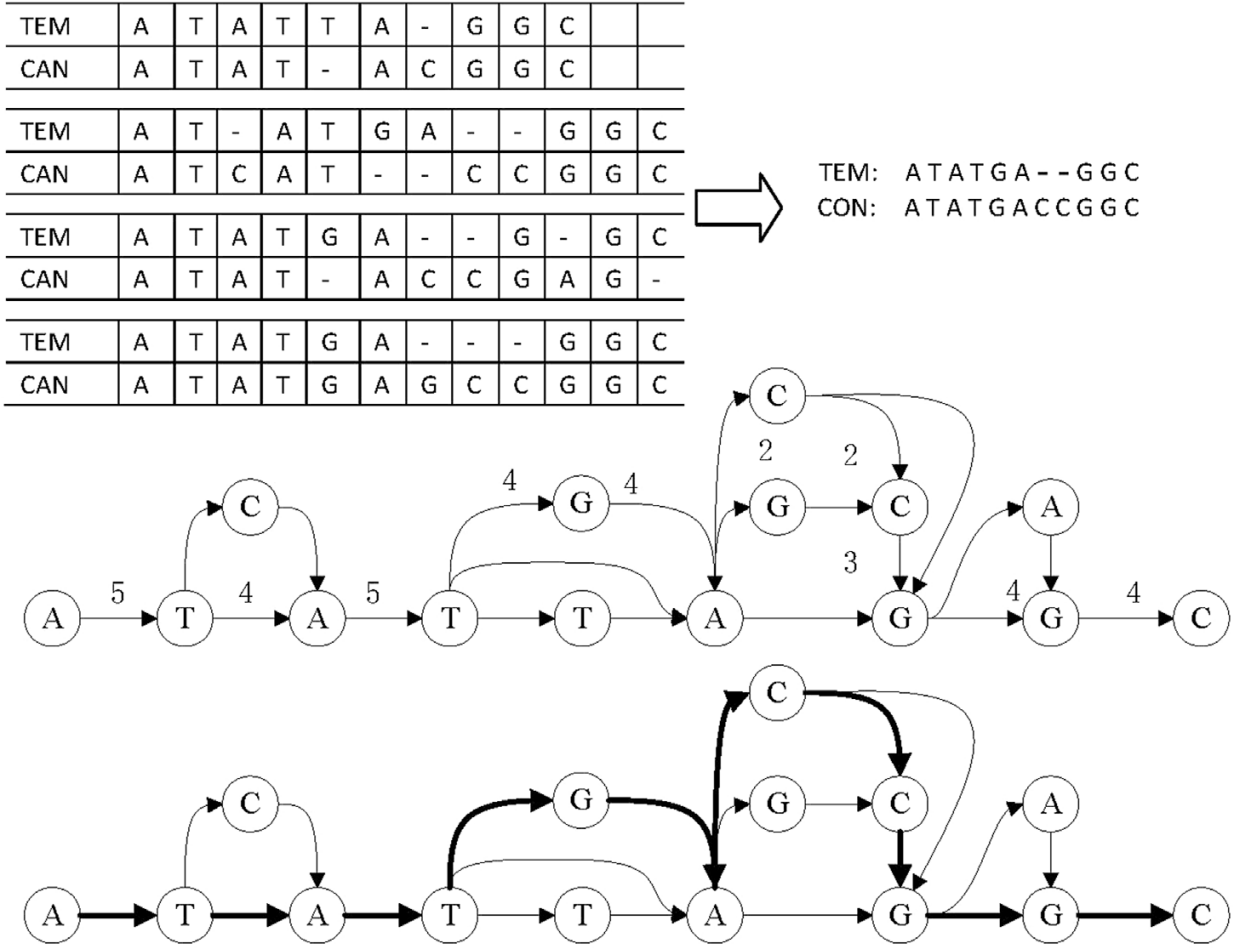
Principle of consensus generation in DAGCon. The bold path in the graph is the maximum weight path, and it is also the path to generate the final consensus sequence.

LCAT adopts DAGCon to get consensus, as shown in Figure 7. DAGCon traverses the candidate reads of complex regions, continuously adds paths to the graph, and selects the path with the largest edge weight as the final consensus sequence. Due to the need for constructing the graph, DAGCon algorithm is slower but has a higher accuracy, which is suitable for complex areas. Usually, the length of the complex area is small, i.e., less than 10 bp, so this step is not time-consuming. The time consumption is much less than using all reads to construct the graph and get the consensus.

#### 2.3.4 Merging error correction results

LCAT combines the consensus results of the simple and complex areas to obtain the final error correction result of the template read.

#### 2.3.5 Implementation of the LCAT algorithm

LCAT software is implemented in C++ for the Linux platform. The input of LCAT is the initial long reads, and the output is the error correction results. LCAT is freely accessible at https://github.com/Xingyu-Liao/LCAT.

## 3 Evaluation

### 3.1 Experimental design

To evaluate the error correction performance of LCAT on third-generation sequencing reads of the transcriptome, we ran LCAT and MECAT on four datasets of long reads from species: *Mouse*, *Zebra finch*, *Calypte anna*, and *Human*. In addition, we compared and analyzed the performance of LCAT and MECAT from two perspectives: basic read properties and transcriptome properties.

### 3.2 Datasets and performance measurements

#### 3.2.1 Datasets

The long reads of *Mouse*, *Zebra finch*, *Calypte anna*, and *Human* were used in our experiments. The mouse and human data are sequenced by Nanopore technology, while zebra finch and *Calypte anna* are sequenced by PacBio technology. Datasets can be downloaded from the NCBI SRA database (https://www.ncbi.nlm.nih.gov/sra/), GitHub (https://github.com/nanopore-wgs-consortium/NA12878/blob/master), and PacBio sequencing platform. Table 1 shows the basic characteristics of the four datasets, including species, read size, sequencing technology, base size, length, read/base mapping rates, and error rates. In addition, the corresponding reference genomes and annotation files were downloaded from the NCBI (https://www.ncbi.nlm.nih.gov/) and Ensembl websites (ftp://ftp.ensembl.org/pub/). The version number of genomes and the annotation files are shown in Table 2.

**TABLE 1:** Details of raw reads.

Type	Mouse	Zebra finch	*Calypte anna*	Human
data_id	ERR2401483	zebra_subreads	anna_subreads	NA12878
Technology	Nanopore	PacBio	PacBio	Nanopore
read_number	740,776	4,812,464	4,144,838	15,152,101
base_number	1,353,969,728	14,168,047,486	11,993,639,660	13,938,188,440
mean_size (bp)	2,011	2,944	2,893.6	932.9
minmum_size (bp)	76	50	50	48
maxmum_size (bp)	98,376	59,135	2,934	16,110
read_map_ratio	86.80%	95.22%	94.35%	97.46%
base_map_ratio	90.95%	86.41%	83.72%	83.49%
error_rate	13.81%	13.36%	12.56%	15.00%
mismatch_rate	3.96%	3.77%	3.31%	4.49%
insert_rate	1.87%	5.91%	5.49%	4.65%
delete_rate	7.99%	3.68%	3.77%	5.86%

**TABLE 2:** Reference genome and annotation files for four species.

Type	Reference genome/annotation file
Mouse	Mus_musculus.GRCm38.dna.primary_assembly.fa
	Mus_musculus.GRCm38.87.gtf
Zebra finch	Taeniopygia_guttata.bTaeGut1_v1.p.dna.toplevel.fa
	Taeniopygia_guttata.bTaeGut1_v1.p.99.gtf
*Calypte anna*	GCF_000699085.1_ASM69908v1_genomic.fna.fa
	GCF_000699085.1_ASM69908v1_genomic.gtf
Human	Homo_sapiens.GRCh38.dna.primary_assembly.fa
	Homo_sapiens.GRCh38.94.gtf

#### 3.2.2 Performance measurements

We aligned the corrected long reads and raw reads to the corresponding reference genomes to assess their quality. The aligner Minimap2 was used for these alignments because it is a typical aligner for transcriptome sequences with fast speed and relatively high sensitivity.

We made a comparative analysis from two perspectives of basic read properties and transcriptome properties using the LR_EC_analyser (Myers, 2014). Basic read properties include the following measurements: 1) *#read* is the number of corrected reads, and *%read* is the number of reads over the total number of raw long reads. *#base* is the number of corrected bases, and *%bases* is the number of bases over the total number of raw long bases; 2) max size is the maximum read size, min size is the minimum read size, and the mean size is the average size of reads; 3) *#umr* is the number of unmapped reads, and *%umr* is the number of unmapped reads over the total number of outputted reads. *#umb* is the number of unmapped bases, and *%umb* is the number of unmapped bases over the total number of outputted bases; 4) error rate is the number of non-matches bases over the number of corrected bases, and mismatch rate, delete rate, and insert rate are the number of mismatch bases, delete bases, and insert bases over the total number of outputted bases, respectively.

Transcriptome properties include the following measurements: 1) we measured the number of genes with reduced, unchanged, and increased number of isoforms to assess the ability of the correction tool to retain isoforms. There are multiple isoforms under the same gene. The smaller the number of genes whose isoform type decreases, the greater the number of genes that remain unchanged and increase after error correction, indicating that the error correction tool has a strong ability to retain isoforms; 2) we counted the loss of transcripts with different relative coverages in the raw reads after error correction to reflect the ability of the error correction tool to retain the isoforms and explained that the error correction tool is biased toward transcripts of different expression levels. A detailed instruction for using the LCAT, as well as the tools, and corresponding commands used during the evaluation is found in Supplementary Sections S1, S2.

## 4 Results

### 4.1 Results of basic read properties

The throughput information after correcting four species is listed in Table 3. For the four datasets, LCAT retained slightly more reads than the MECAT reads or keeps them consistent. The ratio of reads corrected by LCAT to the raw reads is also higher than that corrected by MECAT. In terms of the number and proportion of bases after error correction, LCAT performed better than MECAT in the four species. Both MECAT and LCAT specify the minimum output size of the read in the tool. In this experiment, we set the minimum output size to 100, which is also close to the minimum size of the raw read. The maximum and average size of the reads corrected by LCAT is higher than MECAT. LCAT adopts the read alignment algorithm mecat2pw, which is also used in MECAT, so that the template and candidate reads produced by the two tools are consistent. However, LCAT determines the left and right boundaries by calculating the sum of matching, insertion, and skip and judging whether the sum is greater than the minimum coverage. MECAT does not generate skip types in this process.

**TABLE 3:** Throughput and size of reads after error correction.

Type	Tool	#read	%read (%)	#base	%bases (%)	Min/max/mean
Anna	MECAT	2,419,884	58.383	8,677,586,239	72.352	102/17,276/3,585
	LCAT	2,419,889	58.383	8,753,708,454	72.986	101/17,415/3,617
Zebra	MECAT	2,776,414	57.692	10,093,872,145	71.244	100/22,106/3,636
	LCAT	2,776,418	57.692	10,186,678,335	71.899	100/22,158/3,669
Human	MECAT	3,946,295	26.045	4,927,016,110	35.349	100/9,542/1,249
	LCAT	3,946,366	26.045	4,953,096,049	35.536	100/9,623/1,255
Mouse	MECAT	459,601	62.043	960,574,963	64.482	100/8,510/2,090
	LCAT	460,168	62.120	964,718,126	64.760	100/8,564/2,096

Table 4 shows the unmapped and error rates of reads corrected by LCAT and MECAT. In the mouse dataset, the mapping rate of LCAT is slightly higher than that of MECAT. In the other three datasets, the mapping rate of reads corrected by LCAT is slightly lower than that of MECAT. The unmapped rate of the four species data after using LCAT error correction is higher than that of MECAT. The deletion error of the reads after error correction is the main error type of the reads. After using LCAT, the read error rate is slightly higher than that of MECAT. Among the three types of errors, LCAT retains more mismatch errors relative to MECAT, while insert and delete error types have a lower proportion than those in MECAT. LCAT is improved based on MECAT, which can achieve the purpose of correcting RNA long reads and has improved data throughput and read length.

**TABLE 4:** Number of unmapped reads and error rates after error correction.

Type	Tool	#umr	%umr (%)	#umb	%umb (%)	%err (%)	%mis (%)	%ins (%)	%del (%)
Anna	MECAT	1,075	0.044	497,881,319	5.738	1.220	0.278	0.303	0.639
	LCAT	1,106	0.046	519,338,138	5.933	1.230	0.304	0.304	0.622
Zebra	MECAT	7,038	0.253	284,406,970	2.818	2.049	0.898	0.331	0.820
	LCAT	7,175	0.258	287,569,929	2.823	2.102	1.032	0.301	0.769
Human	MECAT	32,407	0.821	161,109,780	3.270	2.695	0.207	0.119	2.369
	LCAT	32,423	0.822	162,907,329	3.289	2.701	0.341	0.072	2.288
Mouse	MECAT	108	0.023	27,537,416	2.867	4.398	0.214	0.081	4.103
	LCAT	108	0.023	28,082,945	2.911	4.416	0.275	0.041	4.100

### 4.2 Results of transcriptome properties

**FIGURE 8:**
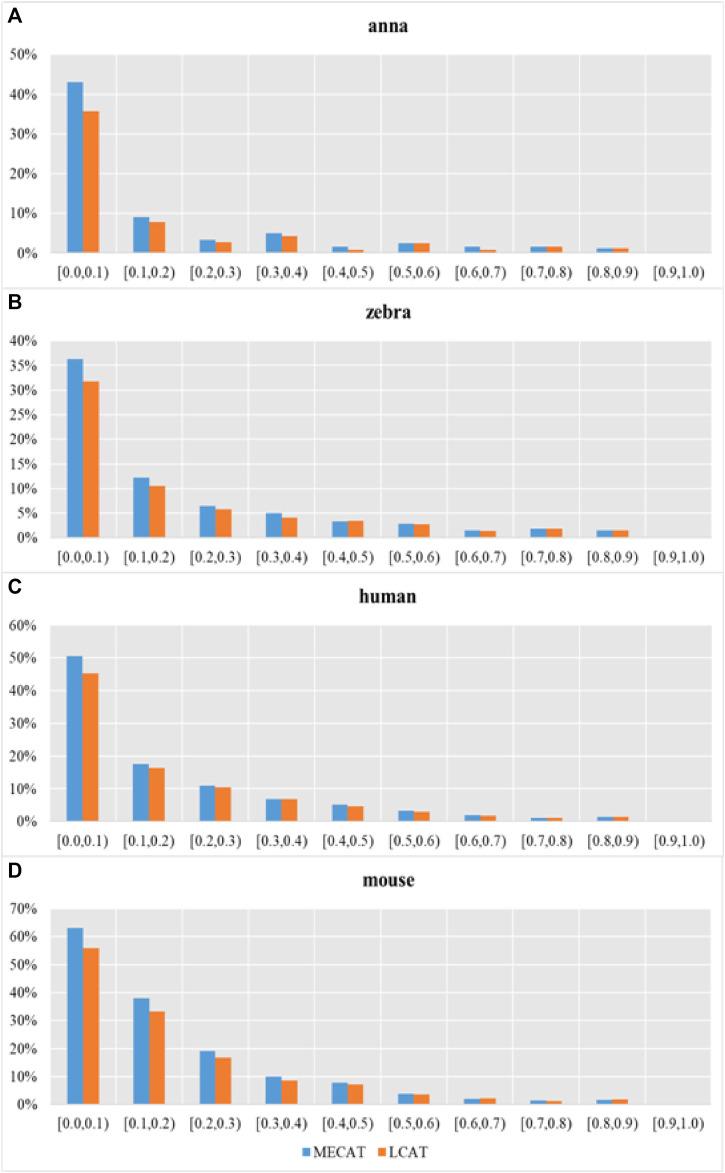
Transcription loss under different relative coverages after correction. Subgraphs **(A–D)** show the number of genes that have undergone isoform species of four species changes after correction using MECAT and LCAT, respectively.

We used evaluation tools such as AlignQC (Jain et al., 2022) and LR_EC_analyser (Myers, 2014), as well as the additional gene annotation file to count the degree of loss of isoform diversity. Table 5 and Figure 8 show the number of genes that have undergone isoform species of four species changes after correction using MECAT and LCAT. The number of isoform changes is the difference between the number of isoforms under each gene in the raw reads and the corrected reads. After error correction using LCAT, the number of genes with reduced isoform species was significantly less than MECAT, while the number of genes with increased isoform species was slightly more than MECAT, and the number of genes with unchanged isoform species under the gene was also significantly more than MECAT. This shows that LCAT can better preserve the diversity of isoforms in genes. We also analyzed the degree of expression of the lost read isoform in the raw read after the error correction tool corrected the error. The relative coverage of transcripts refers to the ratio of the number of the same type of isoforms mapped to the raw read to the number of all transcripts on the gene of this isoform, as shown in Formula (3).

$$relative\_coverage=\frac{isoform\_num}{transcript\_num},$$ (3)

where “*relative_coverage*” represents the value of relative coverage, “*isform_num*” represents the number of isoforms, and “*transcript_num*” indicates the number of transcripts. The low relative coverage rate indicates that the expression level of the transcript in the gene is low and *vice versa*. Table 6 shows the number of transcripts with different relative coverages lost after error correction. The loss refers to the number of transcripts lost after error correction at this relative coverage. Transcripts with low relative coverage have a large number of losses after error correction. This phenomenon is applicable to both LCAT and MECAT. In addition, transcript loss corrected by LCAT is less than MECAT, especially in low relative coverage transcripts.

**TABLE 5:** Number of genes in different isoforms after error correction.

Type	Tool	-(3)	-(2)	-(1)	(0)	+(1)	+(2)	+(3)	Sum
Anna	MECAT	12	34	234	9103	5	0	0	9,388
	LCAT	8	28	207	9153	5	0	0	9,401
Zebra	MECAT	94	303	1,565	8747	39	1	0	10,749
	LCAT	74	263	1,401	8980	48	1	0	10,767
Human	MECAT	4891	1866	2,331	3400	29	1	1	12,519
	LCAT	4334	1920	2,517	3821	43	0	0	12,635
Mouse	MECAT	1,391	1602	2,779	4511	103	2	0	10,388
	LCAT	1,105	1486	2,788	4889	131	11	0	10,410

**TABLE 6:** Loss of transcripts with different relative coverages after error correction.

Type	Tool	0.1	0.2	0.3	0.4	.0.5	0.6	0.7	0.8	0.9	1.0
Anna	MECAT	299	28	6	7	2	3	2	2	2	0
	LCAT	248	24	5	6	1	3	1	2	2	1
Zebra	MECAT	1,888	322	124	66	37	33	14	17	15	8
	LCAT	1,648	280	110	54	38	31	13	17	15	8
Human	MECAT	28,436	2,119	723	269	130	94	33	14	17	13
	LCAT	25,392	1,978	686	267	118	87	32	13	16	17
Mouse	MECAT	8,238	2,240	706	229	119	92	27	16	21	14
	LCAT	7,310	1,967	620	198	110	88	29	14	22	11

**FIGURE 9:**
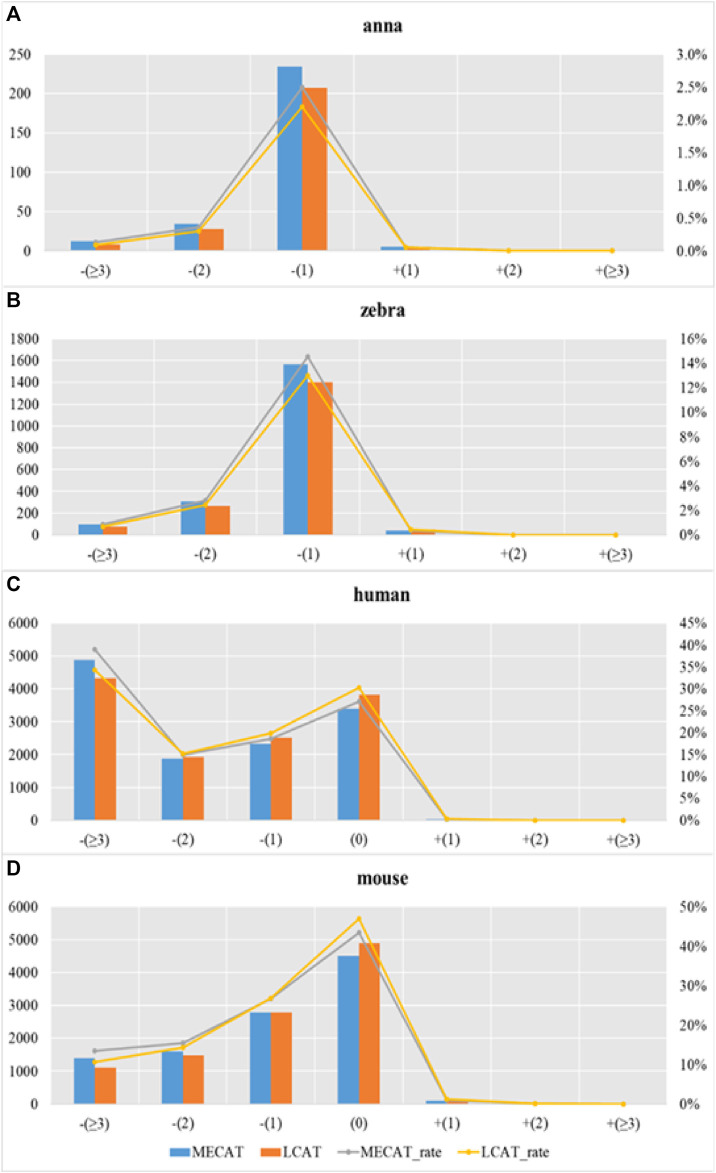
Proportion of transcription loss under different relative coverages to the total number of transcripts with this relative coverage after correction. Subgraphs **(A–D)** show the proportion of transcription loss of four species under different relative coverages to the total number of transcripts with this relative coverage after correction using MECAT and LCAT, respectively.

Figure 9 shows the proportion of transcripts lost under different relative coverages to the total number of transcripts with this relative coverage. During the error correction process, the reads tend to filter the low expression isoforms, and the transcripts corrected tend to be the main isoforms. The LCAT method loses fewer transcripts than MECAT under each relative coverage, and the total number of discarded isoforms is less than MECAT. LCAT is more capable of retaining isoforms than MECAT.

## 5 Conclusion

This study introduces LCAT, a wrapper long-read error correction algorithm for transcriptome sequencing data, to reduce the loss of isoform diversity while keeping MECAT’s error correction performance. LCAT uses the sliding window strategy to filter low identity rate regions with a certain coverage in the alignment step. According to the different categories of the candidate-read areas, the consensus reads are obtained. As a result, LCAT not only improves the quality of reads but also retains the diversity of isoforms, which is more suitable for the error correction of RNA sequencing data. In the future, we will expand this work in the following two directions: 1) the sliding window strategy is combined with other self-correcting algorithms to improve the throughput and accuracy of reads after error correction; 2) the error correction and assembly algorithms are combined to increase the read length and construct more full-length transcripts.

## Data Availability

The datasets presented in this study can be found in online repositories. The names of the repositories and accession numbers can be found in the Supplementary Material.
