# Supplementary material for: LCAT: an isoform-sensitive error correction for transcriptome sequencing long reads
Source: Front Genet. 2023 May 24;14:1166975. doi: 10.3389/fgene.2023.1166975 (PMC10245045; doi:10.3389/fgene.2023.1166975)
Supplement: Supplementary file 1 [file Table1.docx]

Table 1. Details of raw reads

| Type | Mouse | Zebra finch | Calypte anna | Human |
| --- | --- | --- | --- | --- |
| Data id | ERR2401483 | [SRR8551560](https://trace.ncbi.nlm.nih.gov/Traces?run=SRR8551560) | [SRR9184412](https://trace.ncbi.nlm.nih.gov/Traces?run=SRR9184412) | NA12878 |
| Download link | <https://www.ncbi.nlm.nih.gov/sra/?term=ERR2401483> | <https://www.ncbi.nlm.nih.gov/sra/?term=SRR8551560> | <https://www.ncbi.nlm.nih.gov/sra/SRX5956899> | <https://github.com/nanopore-wgs-consortium/NA12878/blob/master/RNA.md> |
| Technology and platform | Nanopore MinION | Pacbio SMRT | Pacbio SMRT | Nanopore MinION |
| Read number | 740,776 | 4,812,464 | 4,144,838 | 15,152,101 |
| Base number | 1,353,969,728 | 14,168,047,486 | 11,993,639,660 | 13,938,188,440 |
| Mean size(bp) | 2,011 | 2,944 | 2,893.6 | 932.9 |
| Minimum size(bp) | 76 | 50 | 50 | 48 |
| Maximum size(bp) | 98,376 | 59,135 | 2,934 | 16,110 |
| Read map ratio (%) | 86.80% | 95.22% | 94.35% | 97.46% |
| Base map ratio (%) | 90.95% | 86.41% | 83.72% | 83.49% |
| Error rate (%) | 13.81% | 13.36% | 12.56% | 15.00% |
| Mismatch rate(%) | 3.96% | 3.77% | 3.31% | 4.49% |
| Insert rate (%) | 1.87% | 5.91% | 5.49% | 4.65% |
| Delete rate (%) | 7.99% | 3.68% | 3.77% | 5.86% |
