# Supplementary material for: LCAT: an isoform-sensitive error correction for transcriptome sequencing long reads
Source: Front Genet. 2023 May 24;14:1166975. doi: 10.3389/fgene.2023.1166975 (PMC10245045; doi:10.3389/fgene.2023.1166975)
Supplement: Supplementary file 2 [file DataSheet1.pdf]

Supplementary materials:

LCAT : An isoform-sensitive error correction for transcriptome sequencing long reads

Wufei Zhu[1], Xingyu Liao[2,*]

[1]Department of Endocrinology, Yichang Central People's Hospital, The First College of Clinical Medical Science, China Three Gorges University, Yichang 443000, P.R. China.
[2]Computer, Electrical and Mathematical Sciences and Engineering Division, King Abdullah University of Science and Technology (KAUST), Thuwal, 23955, Saudi Arabia.

* Corresponding author.
E-mail address: Xingyu_Liao@126.com (Xingyu Liao)

S1. README of LCAT

1) Introduction

LCAT (An isoform-sensitive error correction for transcriptome sequencing long reads) is a wrapper algorithm of MECAT, to reduce the loss of isoform diversity while keeping MECAT's error correction performance. The experimental results show that LCAT not only can improve the quality of transcriptome sequencing long reads, but also keeps the diversity of isoforms.

2) Installation

- **Install LCAT**

```
git clone https://github.com/luckylyw/LCAT.git
cd LCAT
make
cd ..
export PATH=/home/tool/LCAT/Linux-amd64/bin:$PATH
```

After installation, all the executables are found in LCAT/ Linux-amd64/bin.

- **Install HDF5**

```
wget
https://support.hdfgroup.org/ftp/HDF5/releases/hdf5-1.8/hdf5-1.8.15-patch1/src/hd
f5-1.8.15-patch1.tar.gz
tar xzvf hdf5-1.8.15-patch1.tar.gz
mkdir hdf5
cd hdf5-1.8.15-patch1
./configure --enable-cxx --prefix=/home/tool/hdf5
make
make install
cd ..
export HDF5_INCLUDE=/home/tool/hdf5/include
export HDF5_LIB=/home/tool/hdf5/lib
export LD_LIBRARY_PATH=$LD_LIBRARY_PATH:/home/tool/hdf5/lib
```

The header files of HDF5 are in hdf5/include. The library files of HDF5 are in hdf5/lib

- **Install dextract**

```
git clone https://github.com/PacificBiosciences/DEXTRACTOR.git
cp LCAT/dextract_makefile DEXTRACTOR
cd DEXTRACTOR
export PATH=/home/tool/DEXTRACTOR:$PATH
```

Edit line 7 in the dextractor makefile as follows:

```
${CC} $(CFLAGS) -I$(HDF5_INCLUDE) -L$(HDF5_LIB) -o dextract dextract.c sam.c bax.c expr.c DB.c QV.c -lhdf5 -lz
make -f dextract_makefile
cd ..
```

3) Quick Start

LCAT can be used to correct RNA long reads produced by PacBio and Nanopore platforms. The options and commands for processing different types of data are introduced below.

- **Correcting Pacbio Data**

  Step 1: Detect overlapping candidates using lcat2pw

  lcat2pw x 0 -d SRR6238555.fastq -o SRR6238555.fastq.pm.can -w wrk_dir -t 40 -n 100 -a 100 -k 4 -g 0

  Step 2: Correct the noisy RNA reads based on their pairwise overlapping candidates using lcat2cns.

  lcat2cns -x 0 -t 40 -p 100000 -a 100 -l 100 -r 0.6 -c 4 -k 10 SRR6238555.fastq.pm.can SRR6238555.fastq corrected_reads.fastq

- **Correcting Nanopore Data**

  Step 1: Detect overlapping candidates using lcat2pw

  lcat2pw -x 1 -d ERR2401483_proccessed_normalid.fasta -o candidatex.txt -w wrk_dir -t 40 -n 100 -a 100 -k 4 -g 0

  Step 2: Correct the noisy RNA reads based on their pairwise overlapping candidates using lcat2cns.

  lcat2cns -x 0 -t 40 -p 100000 -a 100 -l 100 -r 0.6 -c 4 -k 10 candidatex.txt ERR2401483_proccessed_normalid.fasta corrected_reads.fastq

4) Program Descriptions

The introduction of modules designed in LCAT is shown in the following sections, which also include the options and output format of each module.

- **lcat2pw module**

  Input Format: FASTA/FASTQ files

  Commands:

  lcat2pw [-j task] [-d dataset] [-o output] [-w working dir] [-t threads] [-n candidates] [-g 0/1]

Options

-j <integer> job: 0 = seeding, 1 = align. Default: 0.
-d <string> reads file name.
-o <string> output file name.
-w <string> working folder name, will be created if not exist.
-t <integer> number of cput threads. Default: 1.
-n <integer> number of candidates for gapped extension. Default: 100.
-a <integer> minimum size of overlaps. Default: 2000 if x = 0, 500 if x = 1.
-k <integer> minimum number of k-mer match a matched block has. Default: k= 4 if x = 0; k=2 if x = 1.
-g <0/1> whether print gapped extension start point, 0 = no, 1 = yes. Default: 0.
-x <0/x> sequencing technology: 0 = pacbio, 1 = nanopore. Default: 0.

Output Format

the results are output in can format, each result of which occupies one line and 9 fields:

[A ID] [B ID] [A strand] [B strand] [A gapped start] [B gapped start] [voting score] [A length] [B length]

If the -g option is set to 1, two more fields indicating the extension starting points are given:

[A ID] [B ID] [% identity] [voting score] [A strand] [A start] [A end] [A length] [B strand] [B start] [B end] [B length] [A ext start] [B ext start]

In the strand field, 0 stands for the forward strand and 1 stands for the reverse strand. All the positions are zero-based and are based on the forward strand, whatever which strand the sequence is mapped.

- **lcat2cns module**

Input Format *can* format files.

Commands:

lcat2cns [options] input reads output

Options

-x <0/1> sequencing platform: 0 = PACBIO, 1 = NANOPORE. Default: 0
-t <Integer> number of threads (CPUs)
-p <Integer> batch size that the reads will be partitioned
-r <Real> minimum mapping ratio
-a <Integer> minimum overlap size
-c <Integer> minimum coverage under consideration
-l <Integer> minimum length of corrected sequence
-k <Integer> number of partition files when partitioning overlap results (if < 0, then it will be set to system limit value)
-d <Real> identity threshold

-w <Integer>   slide window length
-m <Real>   minimum coverage rate of modify region
-h   print usage info.

If 'x' is set to be '0' (pacbio), then the other options have the following default values:
-t 1 -p 100000 -r 0.9 -a 2000 -c 6 -l 5000 -k 10 -d 0.65 -w 75 -m 0.05
If 'x' is set to be '1' (nanopore), then the other options have the following default values:
-t 1 -p 100000 -r 0.4 -a 400 -c 6 -l 2000 -k 10 -d 0.65 -w 75 -m 0.05

Output Format

The corrected sequences are given in FASTA format. The header of each corrected sequence consists of three components seperated by underlines:

>A_B_C_D

where A is the original read id,
B is the left-most effective position,
C is the right-most effective position,
D is the length of the corrected sequence,
by effective position we mean the position in the original sequence that is covered by at least c (the argument to the option -c) reads.

S2. Commands and workflow used in evaluations

1) Introduction of evaluation tool

LR_EC_analyser stands for Long Read Error Correction analyser. It is a python script that analyses the output of long reads error correctors, like LoRDEC, NaS, PBcR, proovread, canu, daccord, LoRMA, MECAT, pbdagcon, etc. It does so by running AlignQC (https://github.com/jason-weirather/AlignQC) on the BAMs built by the mapping the output of error correction tools to a reference genome (using for example gmap or minimap2) and parsing its output, and creating other custom plots, and then putting all the relevant information in a HTML report. It also makes use of IGV.js (https://github.com/igvteam/igv.js) for an in-depth gene and transcript analysis.

LR_EC_analyser can be applied to evaluate the extent to which existing long-read DNA error correction methods are capable of correcting long reads. **It not only reports classical error-correction metrics but also the effect of correction on long read connectivity (impacts the inference of transcript structure and exon coupling), gene families, isoform diversity, bias toward the major isoform, and splice site detection.**

2) Usage of the evaluation tool

Command: run_LR_EC_analyser.py

[-h] --raw RAWBAM
[--self <self.bam> [<self.bam> ...]]
[--hybrid <hybrid.bam> [<hybrid.bam> ...]]
--genome GENOME --gtf GTF
[--paralogous PARALOGOUS] [-o OUTPUT]
[-t THREADS]
[--colours <self.colours> [<self.colours> ...]]
[--pdf] [--skip_bam_process] [--skip_alignqc]
[--skip_copying]
Long reads error corrector analyser.

Optional arguments:

-h, --help	show this help message and exit
--raw RAWBAM	The BAM file of the raw reads (i.e. the uncorrected long reads) mapped to the genome (preferably using gmap -n 10 -f samse).
--self <self.bam> [<self.bam> ...]	BAM files of the reads output by the SELF correctors mapped to the genome (preferably using gmap -n 10 -f samse).
--hybrid <hybrid.bam> [<hybrid.bam> ...]	BAM files of the reads output by the HYBRID correctors mapped to the genome (preferably using gmap -n 10 -f samse).
--genome GENOME	The genome in Fasta file format.
--gtf GTF	The transcriptome in GTF file format.
--paralogous PARALOGOUS	A file where the first two columns denote paralogous genes (see file Getting Paralogs.txt to know how you can get this file).
-o OUTPUT	Output folder
-t THREADS	Number of threads to use

--colours <self.colours> [<self.colours> ...]	A list of colours in hex encoding to use in the plots. Colour shading is nice to show related corrections (e.g. full-length, trimmed and split outputs from a same tool),but unless the analysis is on few tools, it is hard to find a nice automated choice of colour shading. Hand-picking is more laborious but produces better results.This parameter allows you to control the colors of each tool. The order of the tools are: raw -> hybrid -> self.The hybrid and self ordering are given by parameter --hybrid and --self.See an example of this parameter in https://gitlab.com/leoisl/LR_EC_analyser/blob/master/scripts/command_line_paper.sh.
--pdf	Produce .pdf files of the plots in the <output_folder>/plots directory.
--skip_bam_process	Skips BAM processing (i.e. sorting and indexing BAM files) - assume we had already done this.
--skip_alignqc	Skips AlignQC calls - assume we had already done this.
--skip_copying	Skips copying genome and transcriptome to the output folder - assume we had already done this.

3) Reference and annotation files for four species used in the evaluation

The long reads of *Mouse*, *Zebra finch*, *Calypte anna*, and *Human* were used in our experiments. The mouse and human data are sequenced by nanopore technology, while zebra finch and Calypte anna are sequenced by PacBio technology (**Table 1**). In addition, the corresponding reference genomes and annotation files were from the NCBI website (https://www.ncbi.nlm.nih.gov/) and the Ensembl website (ftp://ftp.ensembl.org/pub/). The version number of genomes and the annotation files are shown in **Table 2**.

Table 1. Details of raw reads

Type	Mouse	Zebra finch	Calypte anna	Human
data_id	ERR2401483	zebra_subreads	anna_subreads	NA12878
technology	Nanopore	Pacbio	Pacbio	Nanopore
read_number	740776	4812464	4144838	15152101
base_number	1353969728	14168047486	11993639660	13938188440
mean_size(bp)	2011	2944	2893.6	932.9
minmum_size(bp)	76	50	50	48
maxmum_size(bp)	98376	59135	2934	16110
read_map_ratio	86.80%	95.22%	94.35%	97.46%
base_map_ratio	90.95%	86.41%	83.72%	83.49%
error_rate	13.81%	13.36%	12.56%	15.00%
mismatch_rate	3.96%	3.77%	3.31%	4.49%
insert_rate	1.87%	5.91%	5.49%	4.65%
delete_rate	7.99%	3.68%	3.77%	5.86%

Table 2. Reference genome and annotation files for four species

Type	Reference genome/annotation file
mouse	Mus_musculus.GRCm38.dna.primary_assembly.fa
	Mus_musculus.GRCm38.87.gtf
zebra Finch	Taeniopygia_guttata.bTaeGut1_v1.p.dna.toplevel.fa
	Taeniopygia_guttata.bTaeGut1_v1.p.99.gtf
calypte anna	GCF_000699085.1_ASM69908v1_genomic.fna.fa
	GCF_000699085.1_ASM69908v1_genomic.gtf
human	Homo_sapiens.GRCh38.dna.primary_assembly.fa
	Homo_sapiens.GRCh38.94.gtf

4) Specific steps for evaluation

First, use minimap2 to align the original reads and error-corrected reads to the reference genome to obtain the sam files, then use LR_EC_analyser to mark the gene and its isoform structure to which each read belongs according to the sam files and gene and exon information in the genome annotation files.

The number of isoforms of each gene in the original reads and the error-corrected reads were counted separately, and the number of isoform changes was calculated by the difference between the two. By counting the number of genes whose isoforms increase or decrease, the degree of loss of gene isoforms after error correction is reflected.

The running commands of minimap2:

```
./minimap2 -ax map-pb ref.fa pacbio.fq.gz > aln.sam
./minimap2 -ax map-ont ref.fa ont.fq.gz > aln.sam
```

“ref.fa” is the reference file.
“pacbio.fq.gz” is the compressed file of pacbio reads.
“ont.fq.gz” is the compressed file of nanopore reads.

The running commands of samtools:

```
./samtools   view   -bS   -@36   aln.sam > aln.bam
./samtools   sort   -@64   aln.bam > aln.sorted.bam
```

The running commands of LR_EC_analyser

```
python run_LR_EC_analyser.py --genome /data/ref.fa --gtf /data/annotation.gtf --raw /data/aln.sorted.bam -o /output/
```

“ref.fa” is the reference file.
“annotation.gtf” is the genome annotation file.
“aln.sorted.bam” is the bam file after sorted.
